# Effect of Acupuncture on Blood Pressure and Metabolic Profile Among Patients With Essential Hypertension: Protocol of a Randomized Clinical Trial

**DOI:** 10.3389/fcvm.2022.888569

**Published:** 2022-06-21

**Authors:** Pan Zhang, Yalan Chen, Furong Zhang, Hong Pei, Mingsheng Sun, Yuzhu Qu, Jiyao Chen, Ting Du, Xiaoguo He, Fanrong Liang, Weiguo Jia, Mingxiao Yang

**Affiliations:** ^1^College of Acupuncture and Tuina, Chengdu University of Traditional Chinese Medicine, Chengdu, China; ^2^College of Health Preservation and Rehabilitation, Chengdu University of Traditional Chinese Medicine, Chengdu, China; ^3^Department of Chinese Medicine, Geriatric Hospital of Sichuan Province, Chengdu, China; ^4^Department of Rehabilitation Medicine, Xishan People's Hospital, Wuxi, China; ^5^The Center of Gerontology and Geriatrics of West China Hospital, Sichuan University, Chengdu, China; ^6^Department of Medicine, Memorial Sloan Kettering Cancer Center, New York, NY, United States

**Keywords:** essential hypertension, acupuncture, metabolomics, randomized controlled trial, study protocol

## Abstract

Essential hypertension is a polygenic cardiovascular disease that is associated with maladaptive metabolic changes. Acupuncture as a non-pharmacologic intervention is used to lower blood pressure and improve metabolic dysfunction. However, such effects have not been clinically characterized. We will conduct a randomized clinical trial to evaluate the antihypertensive effect of acupuncture among patients with essential hypertension and determine the associated metabolic improvements. This study is a phase II, two-arm, randomized, sham-controlled trial (Trial registration: ChiCTR2100043737), in which biospecimens will be collected for metabolic profiling. A total of 64 patients with a clinical diagnosis of essential hypertension will be randomly assigned to either the acupuncture or the sham acupuncture group in a 1:1 ratio. All participants will receive 10 treatments over 4 weeks, with three sessions per week for the first 2 weeks and two sessions per week for the remaining weeks. The primary outcome is the change of the systolic and diastolic blood pressure measured by the 24-h ambulatory blood pressure monitoring from baseline to 4 weeks. Secondary outcomes include the circadian rhythm of blood pressure, sleep quality measured by the Insomnia Severity Index, cognitive function measured by the Montreal Cognitive Assessment, and others. Fasting blood serum and urine samples will be collected at baseline and 4 weeks for targeted and untargeted metabolomics analysis. We will use the mixed-effects model and other related bioinformatics approaches to analyze the clinical and metabolome data. This metabolomic-based trial will provide important clinical data regarding the efficacy of acupuncture for essential hypertension to better inform evidence-based care delivery for hypertension patients. Moreover, the findings will offer important insights into the mechanism of action of acupuncture for hypertension by revealing its effect on metabolism. The results of this study will be used to inform the design of a statistically powered, multicenter, randomized trial. We will publish the study findings in peer-reviewed journals. The ethical approval of this study has been reviewed and approved by the Sichuan Regional Ethics Review Committee on Traditional Chinese Medicine (ID: 2021KL-006). The outcomes of the trial will be disseminated through peer-reviewed publications.

## Introduction

Essential hypertension (EH), or primary hypertension, characterized by sustained blood pressure elevation, is a complex polygenic cardiovascular disease associated with a high morbidity and mortality rate. Over one billion people worldwide live with EH (1). Epidemiological studies reported that the incidence of EH per year is 31.5% in the high-income countries and 28.5% in the low-income countries (2). Because of genetic predisposition and environmental exposures, EH is linked to maladaptive metabolic changes, such as impaired lipid/glucose metabolism, obesity, and vascular toxin generation (3–5). Emerging evidence suggests that metabolic change is an intermediate element of the pathogenic process for EH, which is associated with major organ damages, including cognition impairment, stroke, atherosclerosis, chronic kidney diseases, and retinopathy (6–8). Systemic treatments are critical to achieving satisfactory blood pressure control (9, 10). Antihypertensive medications are the guideline-recommended first-line treatment for hypertension. Albeit availability, antihypertensives are associated with undesirable side effects and low compliance (11, 12). Therefore, safe and proper treatments are still warranted to improve the safety and patient adherence to the conventional blood pressure management paradigm.

Lifestyle factors including food and diet, body weight, daily activity, and stress emerge as modifiers of blood pressure. Exercise and stress management that directly target lifestyle factors have been shown crucial in blood pressure management and metabolic health (12, 13). Acupuncture as a non-pharmacological intervention is used in clinical practice to treat patients with cardiovascular or metabolic disorders (14, 15). Recent clinical trials demonstrated that acupuncture significantly lowers blood pressure when compared with sham control (16–19). A systematic review study showed that acupuncture used in combination with angiotensin-converting enzyme inhibitor drugs improves the clinical outcomes of blood pressure management than drugs alone (20). Evidence from basic science studies revealed that acupuncture regulates the neuroendocrine system to lower blood pressure and exert metabolism protective effects (21, 22). Given the biological linkage between blood pressure and metabolism regulations, it remains largely unknown about the mechanism of acupuncture on blood pressure regulation and metabolism.

Our pilot studies highlighted that the antihypertensive effect of acupuncture was associated with metabolic improvements in EH patients, possibly through modulating fatty acid metabolism and the citric acid cycle (23–25). Building on these preliminary findings, we conducted a hybrid clinical trial among patients with EH to (1) assess the efficacy and safety of acupuncture vs. sham acupuncture on the reduction of blood pressure, (2) evaluate the effect of acupuncture on EH-related sleep and cognitive symptoms and the quality of life, (3) explore potential clinical factors that are predictive of treatment response to acupuncture such as treatment expectancy, sex dimorphism, traditional Chinese medicine body constitution (TCMBC), and (4) investigate the effect of acupuncture on the targeted and untargeted metabolic profile.

## Methods/Design

### Ethics Approval

The study protocol conforms to the Standard Protocol Items: Recommendations for Interventional Trials (SPIRIT) 2013 statement and standards for reporting interventions in clinical trials of acupuncture (STRICTA) (26, 27). The present study protocol has already been ethically reviewed and approved by the Sichuan Regional Ethics Review Committee on Traditional Chinese Medicine with the ethical approval number: 2021KL-006.

### Study Design

**Figure 1:**
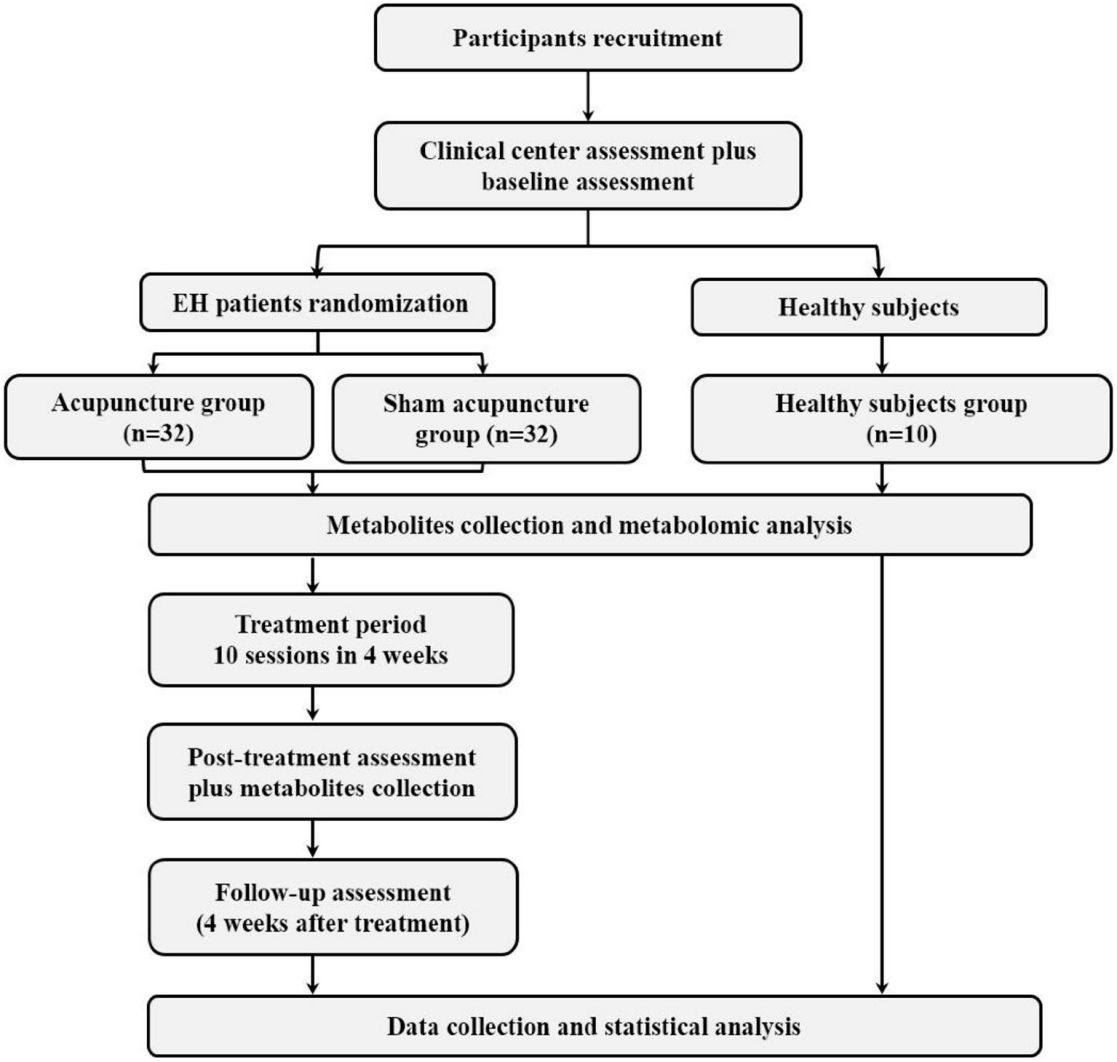
Flowchart of the trial design.

This study is a randomized controlled trial conducted at the Chengdu University of Traditional Chinese Medicine and the Geriatric Hospital of Sichuan Province, Chengdu, China. A total of 64 patients with essential hypertension and 10 healthy subjects will be recruited. 64 patients will be randomly allocated in a 1:1 ratio to either the acupuncture or sham acupuncture group. Patients will receive 10 treatments over 4 weeks. The primary outcome is the 24-h average blood pressure measured with 24-h blood pressure monitoring (24-h ABPM) at 4 weeks. Secondary outcomes include blood pressure measured at 8 weeks, the 24-h circadian rhythm of blood pressure, the sleep quality measured by the Insomnia Severity Index (ISI), cognitive function measured by the Montreal Cognitive Assessment (MoCA), TCMBC, health-related quality of life, patient's treatment expectancy, and adverse events. The details of the study design are shown in Figure 1. The timeline of recruitment, interventions, and outcome measurements is shown in Table 1.

**Table 1:** Trial process chart.

**week**	**-1 week**	**0 week**	**1 week**	**4 weeks**	**8 weeks**	
	**Baseline**		**Treatment period**		**Follow-up period**	
**Enrolment**						
Eligibility test	**√**					
Informed consent	**√**					
Inclusion/Exclusion criteria	**√**					
Demographics		**√**				
Medical history		**√**				
Physical examination		**√**				
Laboratory test		**√**				
Expectation of acupuncture		**√**		**√**		
Random allocation		**√**				
**Interventions**						
Acupuncture group			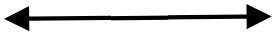			
Sham acupuncture group			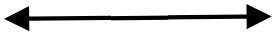			
**Metabolomic analysis**					
Serum metabolites		**√**		**√**		
Urine metabolites		**√**		**√**		
**Outcomes**						
24-h ABPM		**√**		**√**	**√**	
Office blood pressure			**√**	**√**		
TCMBC		**√**		**√**	**√**	
ISI		**√**		**√**	**√**	
MoCA		**√**		**√**	**√**	
SF-12		**√**		**√**	**√**	
**Participants' safety**						
Adverse event	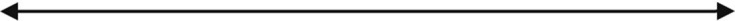					
Safety assessment	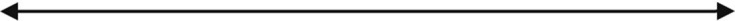					
Causes of dropout				**√**	**√**	
Compliance analysis				**√**	**√**	

### Participants

#### Recruitment Strategies

We will enroll participants from the Chengdu University of Traditional Chinese Medicine, the Geriatric Hospital of Sichuan Province, and their local communities. We will use different recruitment strategies such as posters, leaflets, social media, and free medical consultations to inform and recruit potential patients. We will also enroll patients from physician referral.

#### Eligibility Criteria

##### Hypertension Patients

###### Diagnostic Criteria.

The patient will be diagnosed with essential hypertension according to the 2017 ACC/AHA Clinical Practice Guidelines (16).

###### Inclusion Criteria.

(1) Between 18 to 60 years old, (2) free from any kind of drugs at least 2 weeks before inclusion, (3) naivety to acupuncture treatment for hypertension, and (4) willingness to participate in this study and provide written conformed consents.

###### Exclusion Criteria.

(1) With serious primary diseases such as cardiovascular, cerebrovascular, kidney and hematopoietic system, or metabolic diseases such as diabetes and thyroid dysfunction; (2) with skin diseases and skin damage on the skin or acupoints of the test site; (3) women with pregnancy or lactation or have pregnancy plans in the past year; (4) with history of epilepsy and obstructive sleep apnea syndrome; (5) with history of severe mental disorders or psychoactive substance dependence; (6) with abnormality in a laboratory test of blood biochemistry or with contagious risks; (7) used to suffer from acute diseases in recent 2 weeks, such as high gastritis or fever; (8) once received acupuncture treatment; (9) participating in other clinical trials.

##### Healthy Subjects

Healthy subjects of similar age, body mass index (BMI), and gender will be recruited mainly from the local communities. These participants have no functional or organic disorders, are not suffering from the common cold or other conditions, and have not received acupuncture treatments or taken medications within the past three months. In addition, alcoholics, smokers, and women with pregnancy or lactation will not be included.

#### Patient Enrollment

A cardiovascular physician will confirm the diagnosis of interested participants based on the 24-h ABPM test (A&D Co. Ltd., Japan TM-2430). A clinical research coordinator (CRC) will screen the participant based on the eligibility criteria, and pre-consent for a physical examination and eligibility laboratory tests. The eligibility of an individual who is deemed potentially eligible per CRC will be further confirmed by a study physician. The CRC will consent and register the participant under the patient information system at the Geriatric Hospital of Sichuan Province.

### Randomization

An independent researcher who will not be involved in contact with patients generates the random sequence in R (28). Each random sequence digit will be sealed in an opaque envelope by a CRC. The acupuncture practitioner opens the sealed envelope to learn the group allocation of a participant before the delivery of the initial acupuncture session.

### Blinding

All the participants, outcome assessors, and biostatisticians will be masked to group allocation during the study period. Although acupuncture practitioners will not be masked in this trial, they are not involved in any process of outcome assessment. Furthermore, all treatment sessions and outcome assessments will be conducted in a quiet, separate room for each patient to restrict communications between participants and ensure subject blinding. During treatment, patients will be instructed by acupuncture to lie in a supine position and be covered by a light blanket to enhance blinding.

### Interventions

**Figure 2:**
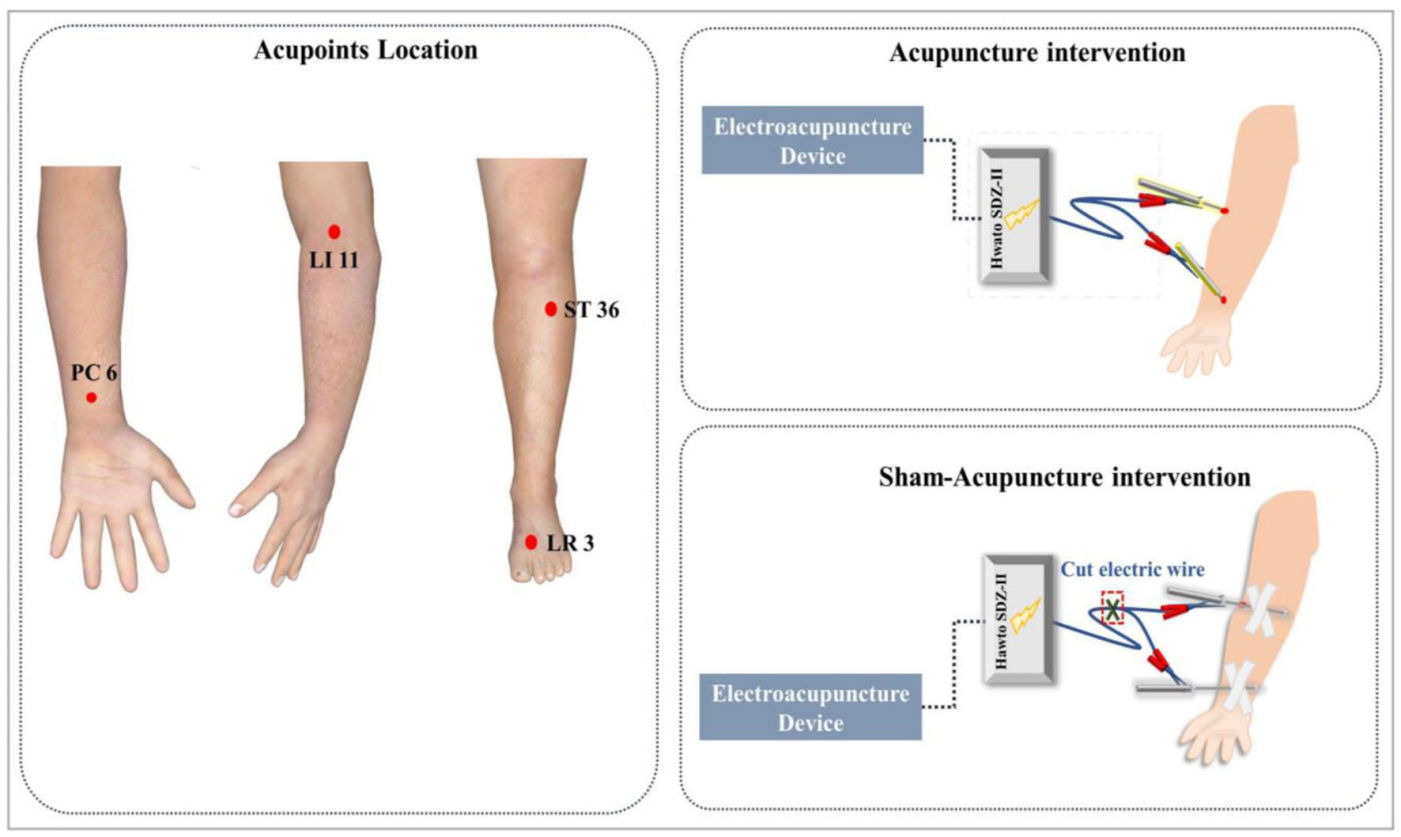
Acupoints Location, acupuncture intervention and sham-acupuncture intervention.

Participants in both groups will receive 10 sessions of acupuncture treatment throughout 4 weeks, three times per week for weeks 1 and 2, and twice per week for weeks 3 and 4. The treatment protocol was developed by consensus with experienced acupuncture physicians and based on our previous studies (20, 25, 29, 30) (Figure 2; ppendix Table 1). All acupuncturists in this trial are registered Chinese Medicine practitioners in China and with at least 3 years of clinical experience in acupuncture practice. Before taking part in this study, all acupuncturists will be required to complete a 40-h standard training course to master the study protocol. When finished, acupuncturists will be required to take an examination to recite the protocol verbally and provide a demonstration of each technique.

#### Acupuncture Intervention

Patients in the acupuncture group will receive electroacupuncture treatment. Sterile disposable acupuncture needles (0.18 × 25 mm, 0.18 × 30 mm; Zhongyan Taihe Medical Instrument Co., Ltd., Beijing, China) will be used for treatment. The manipulations are as follows: Acupuncture needles will be inserted into acupoints at a depth of 20-25 mm after skin disinfection using an alcohol wipe. After insertion, needles will be manually manipulated by gentle lifting, thrusting, and rotating until to achieve the *de-qi* sensation. We will use Hwato therapeutic apparatus (Hwato SDZ-II Acupoint Nerve Stimulator, Suzhou Medical Co., Ltd.) to connect the needles at the two acupoints located in the ipsilateral limb. The frequency will be 60/100 Hz, and the electric current will vary from 1.0 to 2.0 mA until the patients feel comfortable. The acupuncture needles will be retained for 25 min in each session. In addition, all participants will receive lifestyle education on how to improve blood pressure through diet, weight control, and exercise.

#### Sham Acupuncture Intervention

Patients in the sham acupuncture group will receive a non-invasive sham acupuncture treatment (31). Acupuncturists will apply a plastic needle guiding tube on the same acupoints in the arm and leg and flick it to produce some discernible sensation; then, a needle with a piece of adhesive tape will be affixed immediately without skin penetration. We will connect the taped needles with a mock transcutaneous electrical stimulation unit (Hwato SDZ-II Acupoint Nerve Stimulator, Suzhou Medical Co., Ltd.), which will emit a sound and possess a blinking light as the acupuncture group but without any electrical stimulation, for 25 min in each session. The sham treatment will be scheduled at the same course and frequency as the experimental group. Participants in the sham acupuncture group will also receive the same lifestyle education.

### Outcome Measurements

#### Primary Outcome

The primary outcome is the change in mean 24-h systolic and diastolic blood pressure at week 4. The 24-h ABPM is a common and objective measure of blood pressure validated in clinical practice and research, which provides accurate estimates of blood pressure throughout the day (32, 33). We will also measure office blood pressure with a mercury gravity manometer before and after each acupuncture treatment, till 8 weeks.

#### Secondary Outcomes

##### Insomnia Severity Index (ISI)

The ISI is a validated patient-reported outcome of insomnia designed to specifically assess the impact of insomnia on daytime functioning and the severity of associated distress (34). The ISI includes seven items that are scored on a five-point scale ranging from 0 to 4 with higher scores representing more severe insomnia symptoms. The optimal cutoff scores are 0-7 (no clinically significant sleep difficulties), 8-14 (sleep difficulties warrant further investigation), and 15+ (presence of clinically significant insomnia). The ISI has demonstrated internal consistency, reliability, construct validity, specificity, and sensitivity (34). Participants who report a ≥8 reduction in ISI score or achieve an ISI score <8 after treatment are considered insomnia responders (35, 36).

##### Montreal Cognitive Assessment (MoCA)

The MoCA is a rapid screening instrument to identify mild cognitive impairment. The validity and reliability of the MoCA have been demonstrated in previous studies (37, 38). MoCA includes eight cognitive domains to assess attention and concentration, executive functions, memory, language, visuoconstructional skills, conceptual thinking, calculations, and orientation (39). The total possible score is 30 points, and a score of ≥26 is considered a non-cognitive impairment. We will use the MoCA to monitor the effect of intervention on hypertension-related cognitive impairment.

##### The Short Form-12 Health Survey (SF-12)

The SF-12 is a generic and well-established health-related quality of life measure that has been commonly used in both general populations and various patient cohorts (40). The SF-12 contains 8 domains, including physical functioning; role-physical; bodily pain; general health; vitality; social functioning; role-emotional; and mental health (41). The SF-12 can be completed in 2 min. The SF-12 is used to evaluate the treatment effect in patients with hypertension (42).

##### Traditional Chinese Medicine Body Constitution (TCMBC)

TCMBC is a validated tool of patient categorization according to Chinese medicine diagnosis, which has been widely applied in practice by Chinese medicine practitioners (43). TCMBC has 60 items to measure the 9 types of patients: gentleness, Qi-deficiency, Yang-deficiency, Yin-deficiency, phlegm-wetness, wetness-heat, blood-stasis, Qi-depression, and special diathesis (44).

##### Patient's Treatment Expectancy

The Acupuncture Expectancy Scale (AES) is a 4-item instrument developed to measure outcome expectancy in the context of the acupuncture treatment (45). It has demonstrated reliability and validity in oncology populations with a Cronbach α of 0.95 and is sensitive to change over time in response to the acupuncture treatment (46). It has also been shown to correlate with patient self-efficacy and treatment satisfaction (45). The score ranges from 4 to 20, with higher scores indicating a greater patient expectation of improvement.

### Sample Collection and Preservation

We will collect fasting serum and urine samples before acupuncture treatment and on the second day of the final treatment from 8:00–9:00 a.m. We will also collect biospecimens from healthy subjects. Participants are required to (1) be free from the intake of alcohol, refresher drinks, coffee, amino acids, hormone, and vitamin supplements 48 h before sampling, (2) and maintain a regular diet and sleep habits, (3) avoid strenuous exercise 24 h before sampling; and (4) be fasting for 12 h before sampling. Fasting blood samples will be collected into vacutainer tubes with a coagulant; meanwhile, urine samples will be collected in a urinal cup. Both blood and urine samples will be immediately cooled to 4°C. Then these samples will be centrifuged within 2 h at 1500 rpm for 15 min. When completed, serum and urine samples will be separated and transferred to new vials. These samples will be finally stored frozen (−80°C) until metabolomic analysis preparation.

### Metabolomics Analysis

Serum samples will be pre-processed, divided for the available targeted and non-targeted metabolomics methods, and dried. Samples will then be reconstituted and analyzed using metabolomic platforms. The preliminary targeted analysis will consist of mining for metabolites in the Krebs Cycle and others identified in previous studies (23–25). Secondary analysis will leverage non-targeted (differential features analysis) workflows of the entire LC-MS/GC-MS datasets through a combination of the vendor, open source, and commercial software tools, followed by a metabolite identification pipeline to identify statistically important features.

### Safety Assessment

Patient safety will be monitored throughout the trial, with reporting of adverse events (AEs) and serious adverse events (SAEs), such as subcutaneous hemorrhage, vertigo, stroke, or even death. All AEs/SAEs will be recorded carefully and reported immediately in the case report form.

### Sample Size

According to preliminary data from our pilot study, acupuncture was associated with a 9.6-mmHg (δ) greater reduction of 24-h average systolic blood pressure and a 7.7-mmHg greater reduction of 24-h average diastolic blood pressure as compared with sham acupuncture. Conservatively, we hypothesized that acupuncture is associated with a 7.5 mmHg reduction (σ) in 24-h systolic blood pressure compared to sham acupuncture. The significance level was set at 0.05 (α = 0.05, *Z*_α_ = 1.96), and the power of the test was set at 0.9 (1-β = 0.9, *Z*_1−β_ = 1.28). A total of 64 participants will be required in this study (32 in each group), including compensation for an anticipated dropout rate of 20%. Additionally, 10 healthy participants will be recruited as the healthy control group.

### Statistical Analysis Plan

Clinical data will be analyzed with SPSS 24.0 software (SPSS Inc, Chicago, IL, USA). Continuous variables will be presented as mean and SD or as median and interquartile if they are not normally distributed. The baseline variables will be analyzed by the independent-samples t-test and Mann–Whitney U test, where appropriate. Categorical data will be presented as percentages. We will use the mixed-effects model to compare the effect of acupuncture vs. sham acupuncture on blood pressure from baseline to 4 and 8 weeks. The fixed effects are treatment arm, time, group^*^time interaction, and baseline outcome. For metabolomics data, the multivariate and univariate analysis will be performed in combination to determine the plasma metabolites that distinguish EH patients from a healthy subjects, and metabolites that exhibit significant changes before and after treatment in each treatment arm. We will use the Partial least square discriminant analysis (PLS-DA) in SIMCA-P (version 14.0; Umetrics, Umeå, Sweden) and the MetaboAnalyst (http://www.metaboanalyst.ca/MetaboAnalyst/) to fulfill the identification of differential metabolites between groups. All statistical tests will be two-sided, and statistical significance will be set at 5%.

## Discussions

Acupuncture reduces stress and is widely used as a non-pharmacological treatment for cardiovascular diseases and metabolic disorders (20, 47). However, the modulating effects of acupuncture on the cardiovascular and metabolic systems are not well understood so far. Here, we present the design and methodology of our ongoing randomized clinical trial which evaluates the efficacy and safety of acupuncture vs. sham acupuncture on blood pressure and metabolic profile among patients with EH. We will enroll 64 eligible patients and measure their blood pressure with the 24-h ABPM test after administrating 10 acupuncture sessions over 4 weeks. Meanwhile, we will determine the changes in the metabolic profile associated with the treatment effect, via targeted and untargeted metabolic profiling. Additionally, we will identify potential clinical variables and metabolic signatures that are predictive of treatment response to acupuncture among patients with EH. The study findings may have important clinical and research implications.

Till now, the role of acupuncture in blood pressure management remains unclear. Our previous systematic review of randomized clinical trials found inconsistent results concerning the effect of true vs. sham acupuncture on blood pressure, due to the risk of bias associated with sample size, control arms, and treatment population (16, 20, 48). Further, our understanding of the biological foundation of acupuncture is limited, especially in terms of cardiovascular and metabolic modulation. Although metabolic dysregulation has been closely linked to the pathogenesis of EH (49–51), fewer studies have been conducted to assess the metabolic changes in the non-pharmacological blood pressure treatment (23). Our study incorporates metabolomics technology in the design of a rigorous randomized control trial to characterize the metabolic signatures associated with changes in clinical outcomes. This study will provide preliminary data to support the design of a sufficiently powered, randomized controlled trial in the future, which will provide important clinical data to inform the non-pharmacologic management of EH, and physiological information to understand the biological underpinnings of acupuncture for blood pressure and metabolism regulations.

In addition to blood pressure and metabolic profile, this study measures the co-occurring sleep and cognitive symptoms, and health-related quality of life in EH patients, which is seldom addressed by previous studies. Insomnia and sleep disturbances are common and bothersome among EH patients (52). On the other hand, sleep disturbances also contribute to the onset of hypertension and disrupt blood pressure control, resulting in major cardiovascular events like stroke (53, 54). Further, hypertension leads to maladaptation of the cerebral circulation, resulting in dysregulation of cerebral blood flow, microvascular rarefaction, blood-brain barrier disruption, oxidative stress, and impaired neurovascular coupling, while these pathological alterations in cerebral microvessels would damage microvascular structure, network architecture and function, and contribute to the genesis of cerebral microhemorrhages, lacunar infarcts and white matter injury, causing and exacerbating cognitive impairment (55, 56). At present, those symptoms are inadequately monitored and poorly managed in research and clinical practice, leading to decreased quality of life, especially among older adults (57, 58). Our study will shed light on the effect of acupuncture on sleep and cognitive health among EH patients.

This study will also contribute to the identification of potential biomarkers predicting a more pronounced response to acupuncture among EH patients. The pathogenic process of EH is multifactorial; precision treatment targeting a specific signaling pathway or a certain subtype of the population is developed in the conventional treatment paradigm. However, it remains unexplored in non-pharmacologic treatment. Our pilot study showed that specific metabolic pathways are involved in the regulation of blood pressure by acupuncture, which is promising in outcome prediction during the acupuncture treatment (24, 25). Leveraging metabolomics approaches, we will be able to capture metabolic signatures associated with treatment response to acupuncture. Previous studies also linked patient psychosocial characteristics [such as patient expectancy (59)] and patient characteristics [such as Chinese medicine-based body constitution categorization (60)], to the variations in response to acupuncture treatment for high blood pressure. We will further explore potential phenotypic biomarkers that may predict treatment response to acupuncture, which may provide evidence to inform precision non-pharmacologic blood pressure management.

## Ethics Statement

The studies involving human participants were reviewed and approved by the Sichuan Regional Ethics Review Committee on Traditional Chinese Medicine, which belongs to the Regional Ethical Review Committee of Traditional Chinese Medicine in Sichuan province (reference number: 2021KL-006). Written informed consent will be obtained from all participants.

## Author Contributions

MY conceived and designed the study. PZ, FZ, YC, HP, XH, WJ, TD, and FL participated in the design of the study. PZ, YQ, HP, MS, and XH will carry out the acupuncture treatment for patients. In this study, PZ, YQ, YC, and JC screen, assess, and enroll patients. YC and JC are responsible for collecting the data. MY and PZ drafted the manuscript. FZ, YC, and TD revised the manuscript. All authors have read the manuscript and approved the publication of the protocol.

## Funding

This trial was finally supported by the grant from the National Natural Science Foundation of China (No. 81904304), the Young Elite Scientists Sponsorship Program by the CAST (YESS) (No. CACM-2019-QNRC1-04), and the Technical Development Fund of Wuxi City (No. WX18IIAN043). The funding source had no role in the design of this study and will not have any role during its execution, analyses, interpretation of the data, or decision to submit results.

## Conflict of Interest

MY declared that the funding grants supporting the current work were obtained before he joined MSKCC in 2020. The affiliations reflect both the previous and current institutes that he is affiliated with. The remaining authors declare that the research was conducted in the absence of any commercial or financial relationships that could be construed as a potential conflict of interest.

## Publisher's Note

All claims expressed in this article are solely those of the authors and do not necessarily represent those of their affiliated organizations, or those of the publisher, the editors and the reviewers. Any product that may be evaluated in this article, or claim that may be made by its manufacturer, is not guaranteed or endorsed by the publisher.

## Appendix

**Table 1:** Detailed location of acupoints.

**Acupoints**	**Location**
Quchi (LI-11)	Flexing the elbow, on the lateral end of the transverse cubital crease, at the midpoint between Chize (Lu-5) with the lateral epicondyle of humerus.
Neiguan (PC-6)	On the palmar side of the forearm, on the line connecting Quze (PC-3) with Daling (PC-7), 2 cun above the transverse crease of wrist, between the tendons of palmaris longus and flexor carpi radiadis.
Zusanli (ST-36)	On the anterior side of the shank, 3 cun below Dubi (ST-35), one horizontally placed figure distance lateral to the anterior border of the tibia.
Taichong (LR-3)	On the dorsum of the foot, in the depression proximal to the first metatarsal space.
